# Leaf herbivory imposes fitness costs mediated by hummingbird and insect pollinators

**DOI:** 10.1371/journal.pone.0188408

**Published:** 2017-12-06

**Authors:** Alexander Chautá, Susan Whitehead, Marisol Amaya-Márquez, Katja Poveda

**Affiliations:** 1 Departamento de Biología, Universidad Nacional de Colombia, Ciudad Universitaria, Bogotá, Colombia; 2 Department of Entomology, Cornell University, Ithaca, New York, United States of America; 3 Instituto de Ciencias Naturales, Universidad Nacional de Colombia,Ciudad Universitaria. Bogotá, Colombia; Helmholtz Zentrum Munchen Deutsches Forschungszentrum fur Umwelt und Gesundheit, GERMANY

## Abstract

Plant responses induced by herbivore damage can provide fitness benefits, but can also have important costs due to altered interactions with mutualist pollinators. We examined the effects of plant responses to herbivory in a hummingbird-pollinated distylous shrub, *Palicourea angustifolia*. Through a series of field experiments we investigated whether damage from foliar herbivores leads to a reduction in fruit set, influences floral visitation, or alters floral traits that may influence pollinator preference or pollinator efficiency. Foliar herbivory by a generalist grasshopper led to reduced fruit set in branches that were directly damaged as well as in adjacent undamaged branches on the same plant. Furthermore, herbivory resulted in reduced floral visitation from two common hummingbird species and two bee species. An investigation into the potential mechanisms behind reduced floral visitation in induced plants showed that foliar herbivore damage resulted in shorter styles and lower nectar volumes. This reduction in style length could reduce pollen deposition between different floral morphs that is required for optimal pollination in a distylous plant. We did not detect any differences in the volatile blends released by damaged and undamaged branches, suggesting that foliar herbivore-induced changes in floral morphology and rewards, and not volatile blends, are the primary mechanism mediating changes in visitation. Our results provide novel mechanisms for how plant responses induced by foliar herbivores can lead to ecological costs.

## Introduction

Plants interact simultaneously with diverse communities of antagonistic and mutualistic organisms. Although these interactions are often studied in isolation, plant traits that influence multiple interactions are common, and thus plant responses to one organism can mediate interactions with multiple other organisms [1,2]. For example, herbivore damage can induce complex changes in plant primary and secondary metabolism that reduce subsequent herbivore damage [3], but these changes can also negatively affect interactions with mutualists, including pollinators, seed dispersers, and mycorrhizal fungi [4–6]. The negative effects of induced plant defenses on mutualistic interactions are examples of ‘ecological costs of defense’, which are costs that arise when the expression of defense traits alters interactions between plants and their environment in a way that reduces plant fitness [7]. Ecological costs can occur due to a variety of different mechanisms and may be even more important than physiological costs in determining the overall fitness consequences of defense responses [8]. Here we focus on costs of foliar herbivory that can result from a reduction in the visitation of pollinators.

A growing body of literature has documented an effect of herbivory on plant-pollinator interactions [9]. Altered plant-pollinator interactions can lead to ecological costs when herbivory alters floral traits in a way that either reduces pollinator preference or the efficiency of pollen transfer and ultimately plant fitness. There are now several examples of insect pollinators, including bees and syrphid flies, that avoid flowers on plants that are damaged by herbivores [10–12]. However, there are also examples of plants that become more attractive to pollinators after induction by root herbivores [13]. The effects of herbivory on vertebrate pollinators are less well-documented, but at least one study found a reduction in hummingbird visitation to flowers following experimental defoliation treatments in a tropical herb [14]. In addition to effects on pollinator preference, a few studies have also suggested that herbivory can reduce the efficiency of pollen transfer, either through changes in the duration of visits [10] or changes in floral morphology that reduce the probability that pollinators will contact the sexual organs of the flower effectively [15]. However, we still have only a limited knowledge of the variety of mechanisms through which plant responses to herbivore damage may influence pollination success.

Induced responses to herbivory could be affecting floral traits and pollination through at least three different mechanisms. First, foliar herbivory can reduce the resources available for floral structures or rewards. This may occur because photosynthetic capacity is reduced when leaf tissue is lost or because an increased proportion of available resources are allocated to defense-related processes [16]. The result can be altered flowering phenology, reduced flower number, decreased flower size, or reduced quality and quantity of floral rewards, and any of these changes could make flowers less attractive to pollinators [9,15,16]. Furthermore, many of these changes in floral traits could reduce pollinator efficiency. For example, changes in corolla tube size and morphology are known to influence whether pollinators make effective contact with sexual parts [17,18]. A second mechanism through which foliar herbivory can affect pollination is by inducing the production of deterrent or toxic secondary metabolites, which can be expressed in nectar or pollen and alter pollinator behavior [1,19]. For example, herbivory has been shown to increase the concentration of toxic alkaloids in nectar [1] and this increase in alkaloids negatively affects male plant reproduction [19]. However, evidence has also shown that an increase in alkaloids can be beneficial for the plant by increasing the number of visitors per nectar volume [20]. A third mechanism through which herbivory can influence pollination is through changes in the volatile profiles of flowers [16,21]. Changes in plant volatile production following herbivory are common and can provide important benefits to the plant by deterring subsequent herbivores or attracting natural enemies [22,23], but altered volatile profiles can also disrupt pollinator attraction [4,21]. Although the importance of floral volatile cues has primarily been studied for insect pollinators, some evidence suggests that certain floral volatiles can also alter hummingbird behavior, either increasing or decreasing time of visitation depending on the compounds involved [20].

This study investigates the consequences of foliar herbivory in a hummingbird-pollinated tropical distylous plant: *Palicourea angustifolia* Kunth (Rubiaceae). We hypothesized that foliar herbivory reduces plant fitness by altering plant traits that are essential in pollinator attraction and reward. Very little is known about the natural history of this species, so we first conducted an experiment to describe its pollination requirements at the study site, providing essential background for understanding potential ecological costs. Our primary objective was to determine whether damage from foliar herbivores leads to a reduction in fruit set. After finding this effect we conducted a follow up experiment to assess whether damage from leaf herbivores influences floral visitation by different classes of hummingbird and insect pollinators. Finally, to had better understand the potential mechanisms that could be driving the pollinator-mediated effects of leaf herbivory on fruit set, we hypothesized that herbivory could influence a) floral morphology, b) nectar volume and nectar concentration, and/or c) volatile organic compounds (VOCs) could explain the found patterns.

## Materials and methods

### Study site and system

**Fig 1:**
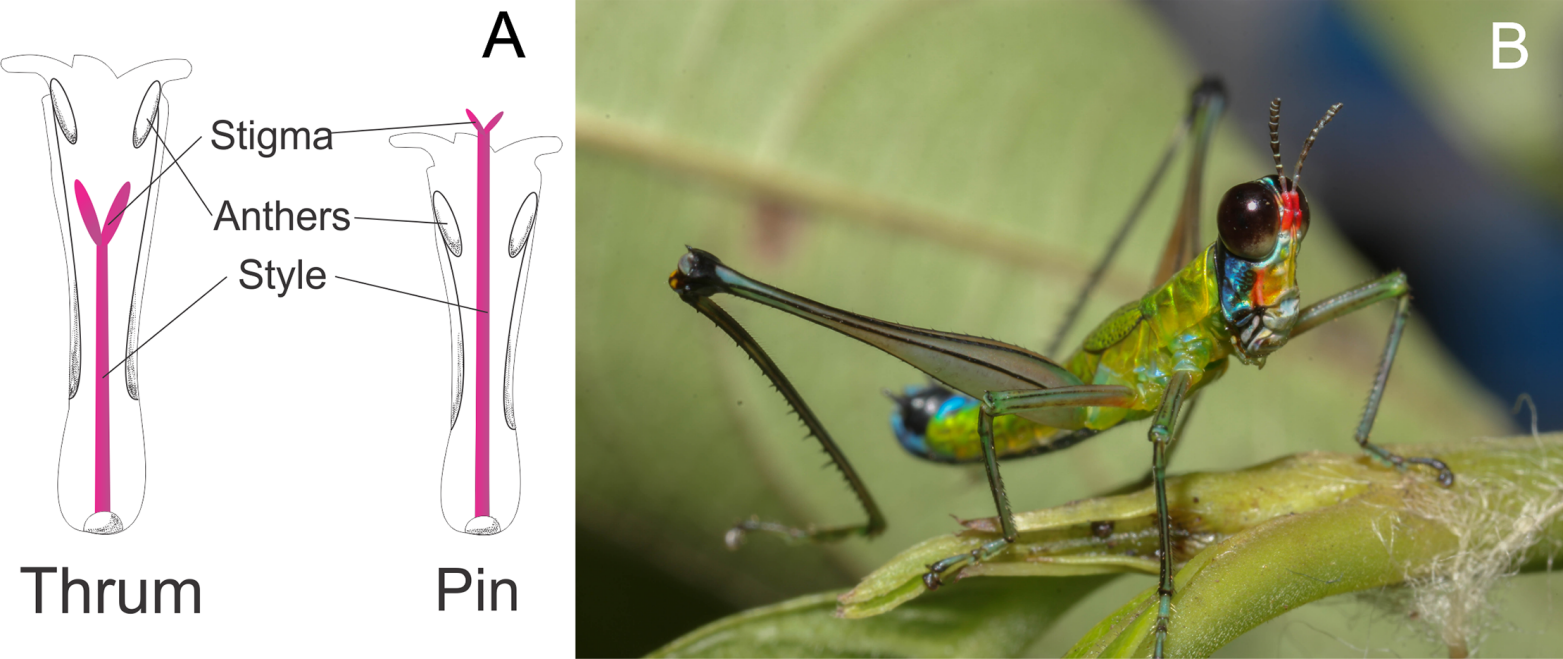
**A. Diagram of thrum and pin floral morphs in *Palicourea angustifolia*. B. *Zeromastax selenesii* feeding on *Palicourea angustifolia* leaves**.

The study was conducted in an Andean forest relict located at 2200 meters in the municipality of El Rosal (Cundinamarca), near the village La Hondura, Colombia between August 18, 2012 and January 24, 2014. The average annual temperature is 14°C, average relative humidity is 85%, and average precipitation is 1410mm. Our study plant, *P*. *angustifolia*, is a common understory shrub of 1.5 to 3.5 m height that is widely distributed between 500-3350m [24]. The inflorescences are terminal panicles with 40 to 160 pink flowers (Chautá pers. obs.). The flowers in the genus *Palicourea* have well developed tubes, and are assumed to be pollinated primarily by hummingbirds [25–27]. Flowers are distylous—approximately 47% of plants are pin morphs with long styles and short stamens and 53% are thrum morphs with short styles and long stamens (Fig 1A, S1 File). This is a common polymorphism that has evolved repeatedly in at least 25 angiosperm families and is often associated with reproductive incompatibility among individuals of the same morph [28]. Nectar volume and nectar concentrations do not differ between the two morphs (S1 File), and both morphs are commonly visited by both hummingbirds and insects (primarily bees, but also wasps and butterflies; Chautá pers. obs.).

For all experiments, the generalist grasshopper *Zeromastax selenesii* (Orthoptera: Eumastacidae, Fig 1B) [29] was used as an herbivore, since it was the main foliar herbivore that we found feeding on *P*. *angustifolia* in the study region. These grasshoppers will consume both leaves and flowers of *P*. *angustifolia*, but floral herbivory was prevented in all experiments (see below). For all experiments, we collected adult *Z*. *selenesii* in the field from *P*. *angustifolia* and other plants on the same day that we began the experiments. Although both adult and juvenile grasshoppers feed on *P*. *angustifolia* we used only adults in the experiments to minimize variation in consumption rates among individuals.

### Pollination requirements

In order to understand the potential for ecological costs mediated by pollination in this system, we first conducted an experiment to determine the dependence of *P*. *angustifolia* on pollinators to successfully set fruit. We chose plants from both pin morphs (N = 15 plants) and thrum morphs (N = 17 plants) for a pollination experiment that assessed whether plants can produce fruits asexually and whether there are differences in fruit production between homomorphic and heteromorphic crosses. On each plant, branches with buds and no open flowers were covered with 'Breather Sleeves' (Palm Tree Packaging, Inc, Florida, USA) to exclude natural visitation from pollinators. When flowers opened, we chose five flowers from each inflorescence and applied one of each of the following five treatments: 1) Manual homomorphic outcrossing (thrum x thrum and pin x pin); 2) Manual heteromorphic outcrossing (thrum x pin and pin x thrum); 3) Manual self-pollination where pollen from the same flower was transferred from the anthers to the stigma; 4) Spontaneous self-pollination where the flowers were bagged during anthesis; and 5) Parthenocarpy test where anthers were removed when the flowers opened. After applying the treatments, the inflorescences were re-bagged to prevent natural visitation and pollen transfer. We monitored development on all inflorescences and recorded whether each flower successfully developed into a fruit.

### Effect of herbivory on fruit set

To determine the effects of herbivory on fruit set, we experimentally manipulated damage 39 plants, each of which was paired with an undamaged control. Pairs of plants were always chosen that were located within two meters of one another to allow for simultaneous observations of pollinators (see below). In some cases, in order to allow for the maximum number of plant pairs located in close proximity to one another, pairs of plants were selected where the two individuals were of different morphs; however, preliminary data showed no differences in visitation rates between pin and thrum plants and we varied the identity of the control plants (pin or thrum) among pairs to avoid any potential bias (t-test = 1.08, p = 0.28). On one plant in each pair we selected and bagged two branches with inflorescences at the bud stage that were at approximately the same size, phenological stage, and height on the plant. One of the branches received herbivore damage where one individual adult of *Z*. *selenesii* was placed inside the bag for three days to consume the leaves. This branch was used to measure locally-induced responses to herbivory. The second branch on the same shrub was bagged in the same manner but received no damage and was used to measure systemically-induced responses to herbivory. In order to prevent the grasshopper from eating the inflorescence, the inflorescence was covered with a second bag during the three days the herbivore was feeding. Damage to leaves varied from approximately 4–49% tissue removed. In rare cases where the grasshoppers died during the three-day damage treatment, plants were excluded from the analysis. The second plant in each pair served as an absolute control in which a single branch that was approximately the same size and height was bagged in the same way without placing any herbivore on the plant. After the three-day herbivore treatments, we removed the grasshoppers and the bags from all branches and allowed natural pollinator visitation to the inflorescences. We counted the total number of flowers that were aborted and the number that successfully set fruit.

### Effects of herbivory on floral visitors

To determine if foliar herbivory affected pollinator visitation, we monitored pollinator visits to 26 pairs of plants from the experiment described above. The locally-induced, systemically induced, and control branches from a single plant pair were then observed simultaneously by one observer to record the identity of the floral visitors (hummingbirds, bees, wasp and butterflies) and the number of flowers visited by each individual visitor during a period of 30 minutes. When pollinator abundance was low or when very few flowers were available on experimental branches, the observations were repeated one or two times on different days to increase the accuracy of the recorded effect. All observations were conducted between October 12, 2013 and 24 January 24, 2014 during sunny or partially overcast weather from 8h to 15h. Just prior to each observation, we counted the number of open flowers on each inflorescence from each of the three branches. Birds were identified to species directly in the field based on the Hilty and Brown bird field guide [30] and insect visitors were collected and identified to species or genus in the lab with help of bee specialist Diana Obregon.

### Effects of herbivory on floral morphology and nectar rewards

To establish if foliar herbivory causes changes in floral morphology and rewards that could alter floral visitation, we selected another 30 pairs of plants (15 pin and 15 thrum) of *P*. *angustifolia*. On each pair, we selected branches with inflorescences at the bud stage to serve as locally-induced, systemically-induced, and control branches and applied grasshopper herbivory treatments as described above. After 3 days, grasshoppers and bags were removed and once the flowers opened we took measurements of corolla length, anther length, style length, and distance from anthers to stigma for one flower randomly chosen from each inflorescence. From a subset of the same inflorescences (5 pin and 6 thrum), we also collected nectar on one randomly chosen flower. Nectar volume was measured using 2μL microcapillary pipettes (Drummond Scientific Company, Broomall, PA). The nectar concentration was estimated using a handheld refractometer (Reichert Digital Brix/RI-Chek). Due to low nectar volumes, all samples were diluted in 0.1 mL distilled water prior to taking nectar concentration measurements. The nectar concentration of the floral nectar was then calculated as the measured solution concentration multiplied by the ratio of the final solution volume to the collected nectar volume.

### Effects of herbivory on volatile emissions of flowers and leaves

To establish if foliar herbivory causes changes in volatile emissions that could alter visitation, we chose ten pairs of plants and applied the treatments of local induction, systemic induction and control as described above. After removing the grasshoppers, we collected volatiles following the headspace method by Kessler and Halitschke (2009) [21] by enclosing single leaves and inflorescences of each branch in 500 mL polyethylene cups fitted with ORBO-32 charcoal adsorbent tubes (Supelco, Bellefonte, PA, USA). Air was pulled through the cup at a flow rate of approximately 250 mL/min for 8 hours using a 12 V vacuum pump (GAST®, Gast Manufacturing Inc., BentonHarbor, MI, USA). Tubes were then capped and kept frozen prior to analysis. Prior to elution, we added tetraline (450 ng) dissolved in toluene (5 μL) as an internal standard to each tube. The tubes were then desorbed with dichloromethane (350 μL) and samples were analyzed by GC-MS [31]. We used a Varian CP-3800 GC coupled with a Saturn 2200 MS and fit with a DB-WAX column programmed as follows: injector at 225°C, temperature initial in the column at 45°C for 6 min, increased at 10°C min−1 to 130°C, increased at 5°C min−1 to 180°C, increased at 20°C min−1 to 230°C, held for 5 min, increased at 20°C min−1 to 250°C, and held for 5 min. Helium carrier gas flow was set to 1 ml min−1 with an electronic pressure control unit. Spectra were collected at −70 eV [31]. Total ion chromatograms were integrated and peak areas of individual compounds were normalized by the area of the internal standard [21].

### Statistical analysis

#### Pollination requirements

To evaluate the differences in fruit production between homomorphic and heteromorphic crosses, we used Fisher’s exact tests for 2x2 contingency tables comparing the binomial counts of successful and unsuccessful fruit set between treatments 1 and 2. These were conducted separately for pin and thrum plants. *P*. *angustifolia* contained two seeds per fruit for all the inspected fruits. Therefore, fruit set was considered as a reliable proxy for seed set. Given that no fruits developed from manual self-pollination, spontaneous self-pollination, or parthenocarpy treatments (see results), no statistical analyses were conducted for these data.

#### Effect of herbivory on fruit set

To determine if there were differences in fruit set among locally-induced, systemically-induced, and control inflorescences, we used a generalized linear mixed model (glmm) with a binomial distribution and the logit link function. The response variable was the binomial count of the number of flowers that developed into fruits and the number that were aborted. Treatment was included as a fixed effect, and the plant pair was included as a random effect. Significant effects of treatment on fruit set were detected through the comparison of models with and without the fixed effect term [32]. Based on a significant effect of treatment (see results), the glmm was followed by a Tukey multiple comparisons test to examine pairwise differences among treatments. These and all subsequent analyses described below were conducted in R version 3.1.2 [33] using the packages lme4 [34] and multcomp [35].

#### Effects of herbivory on floral visitors

To assess the effects of herbivory on pollinator visitation, we first compared total number of visits and the total number of visitors among the three treatments using two separate generalized linear mixed models (glmms) with poisson distributions. The response variables were the total number of visits or visitors summed across all observation periods for each replicate plant pair. Treatment, the number of open flowers, and their interaction were included as fixed effects and plant pair was included as a random effect for both analyses. Next, to further examine the effect of herbivory on individual pollinator species, we summed the total number of visits from each visitor species for each treatment and used a chi-square test of independence to determine whether the frequency distribution of visits among locally-induced, systemically-induced, and control inflorescences differed among the different species of pollinators visiting *P*. *angustifolia*. Based on a lack of independence between treatment and pollinator identity (see results), we analyzed the effect of treatment separately for individual pollinator species. These analyses were only conducted for pollinator species that visited inflorescences in at least five replicate plant pairs. These included just two hummingbird species (*Ocreatus underwoodii* and *Colibri coruscans*), and two bee species (*Trigona fulviventris* and *Paratrigona* sp.). Prior to analysis, we eliminated data from all replicate pairs in which the pollinator species being analyzed did not visit any of the three inflorescences. The total number of visits by the pollinator species being analyzed was summed by replicate pair (total from all observation periods) and we analyzed the effects of treatment on the number of visits using a generalized linear mixed model (glmm) with a poisson distribution. We also included the number of open flowers on the inflorescence (averaging across all observation periods) as a covariate. Due to limited sample sizes in these analyses, we did not include the interaction term between treatment and the number of open flowers. Plant pair was included as a random effect in all analyses. When significant effects of treatment were detected, the glmms were followed by a Tukey multiple comparisons test to determine pairwise differences among treatments.

#### Effects of herbivory on floral morphology and nectar rewards

To assess differences in floral morphology and rewards, we used linear mixed models with treatment, floral morph (pin or thrum), and the interaction between treatment and morph as fixed effects and the replicate plant pair as a random effect. These were conducted separately for the following response variables: corolla length, anther length, style length, anther/stigma distance, nectar volume, and nectar concentration. Nectar concentration data was log-transformed prior to analysis to fit assumptions of normality. Significance of the explanatory variables was determined by model comparisons as described above and followed by Tukey HSD post-hoc tests where appropriate.

#### Effects of herbivory on volatile emissions of flowers and leaves

To examine differences in volatile blends among treatments, we used a random forest (RF) between-group classification algorithm as described in Ranganathan and Borges [36]. This analysis provides several valuable results for understanding differences in complex traits such as volatile blends, including: 1) a proximity matrix of individual samples that can be visualized using a multi-dimensional scaling (MDS) plot, 2) a ranking of the relative importance of different predictor variables (here the individual compounds) in distinguishing among treatment groups, 3) a measure of the prediction accuracy with which unknown samples can be correctly classified into groups using the predictor variables. Tentative identification of the peaks was made using the NIST mass spectrum data base (S2 Table). We conducted the RF analysis in several stages using the packages randomForest [37] and varSelRF [38] in R version 3.1.2 [33]. First, we conducted an RF classification analysis for all six groups (locally-induced, systemically-induced, and control for both leaves and flowers) and visually examined whether there were differences among these groups using an MDS plot. Based on these results, we conducted further analyses to compare the volatile emission of leaves and flowers, the volatile emission among treatments just for leaves and the volatile emission among treatments just for flowers. We used 200 bootstrap iterations of each analysis to select compounds that best distinguished among groups. Compounds that were selected in greater than 20% of bootstrapped models were retained for use in MANOVAs comparing the concentrations of the selected compounds among groups. Where significant overall differences among groups were detected, we followed the MANOVAs with ANOVAs comparing the concentrations of individual compounds among groups.

## Results

### Pollination requirements

Our results suggest that *P*. *angustifolia* is an obligate outcrosser (Table 1). Flowers that received the manual self-pollination, spontaneous pollination and parthenocarpy treatments did not produce any fruits, showing that *P*. *angustifolia* is not self-compatible. In addition, *P*. *angustifolia* exhibited partial heteromorphic incompatibility—pollination was over four times more effective when crossed between pin and thrum morphs. Contingency tables for both pin flowers (*Χ*^2^ = 7.12, df = 1, *P* = 0.0076) and thrum flowers (*Χ*^2^ = 5.56, df = 1, *P* = 0.018) showed that the frequency distribution between homomorphic and heteromorphic crosses were different from a null expectation of 50:50.

**Table 1:** Percentages of fruit production in *Palicourea angustifolia* with six different pollination treatments on pin and thrum flowers. *P*. *angustifolia* produce I higher amount of fruits when the pollen comes from plants of the opposite morph.

Treatment	Pin	Thrum
Homomorphic outcrossing	20	23
Heteromorphic outcrossing	93.3	82.2
Manual self-pollination	0	0
Spontaneus self-pollination	0	0
Parthenocarpy	0	0

### Effects of foliar herbivory on fruit set

**Fig 2:**
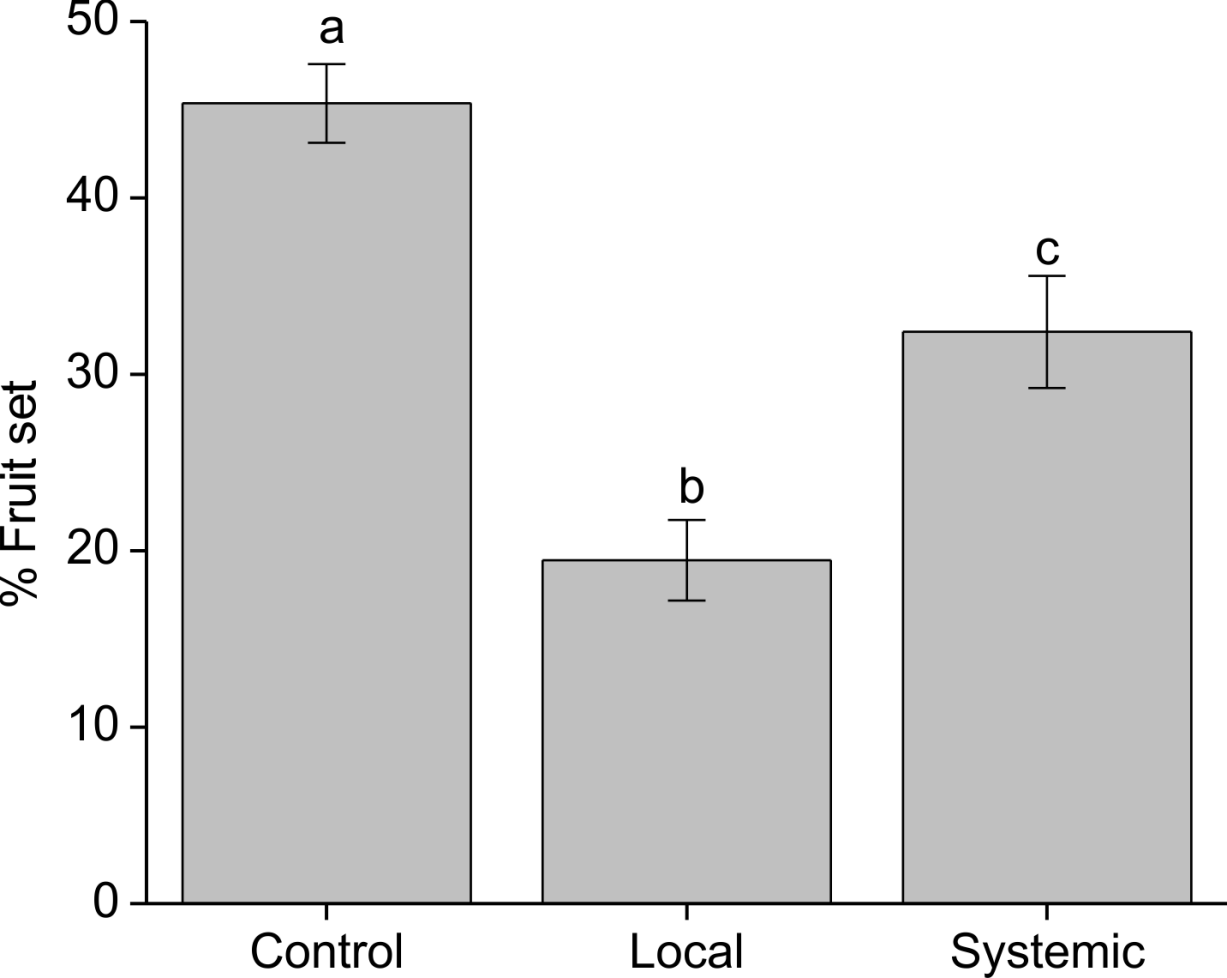
Percentage of fruit set on *Palicourea angustifolia* plants that have been locally or systemically exposed to herbivory by *Zeromastax selenesii* and non-exposed (control plants). Treatments with same letter are not significantly different (Tukey test, P > 0.05).

Fruit set was negatively affected by foliar herbivory, with the highest average fruit set (45%) on control branches, intermediate fruit set (32%) on systemically-induced branches, and the lowest fruit set (19%) on locally-induced branches (*Χ*^2^ = 472.12, df = 2, *P* = <0.001; Fig 2).

### Effects of foliar herbivory on floral visitors

**Fig 3:**
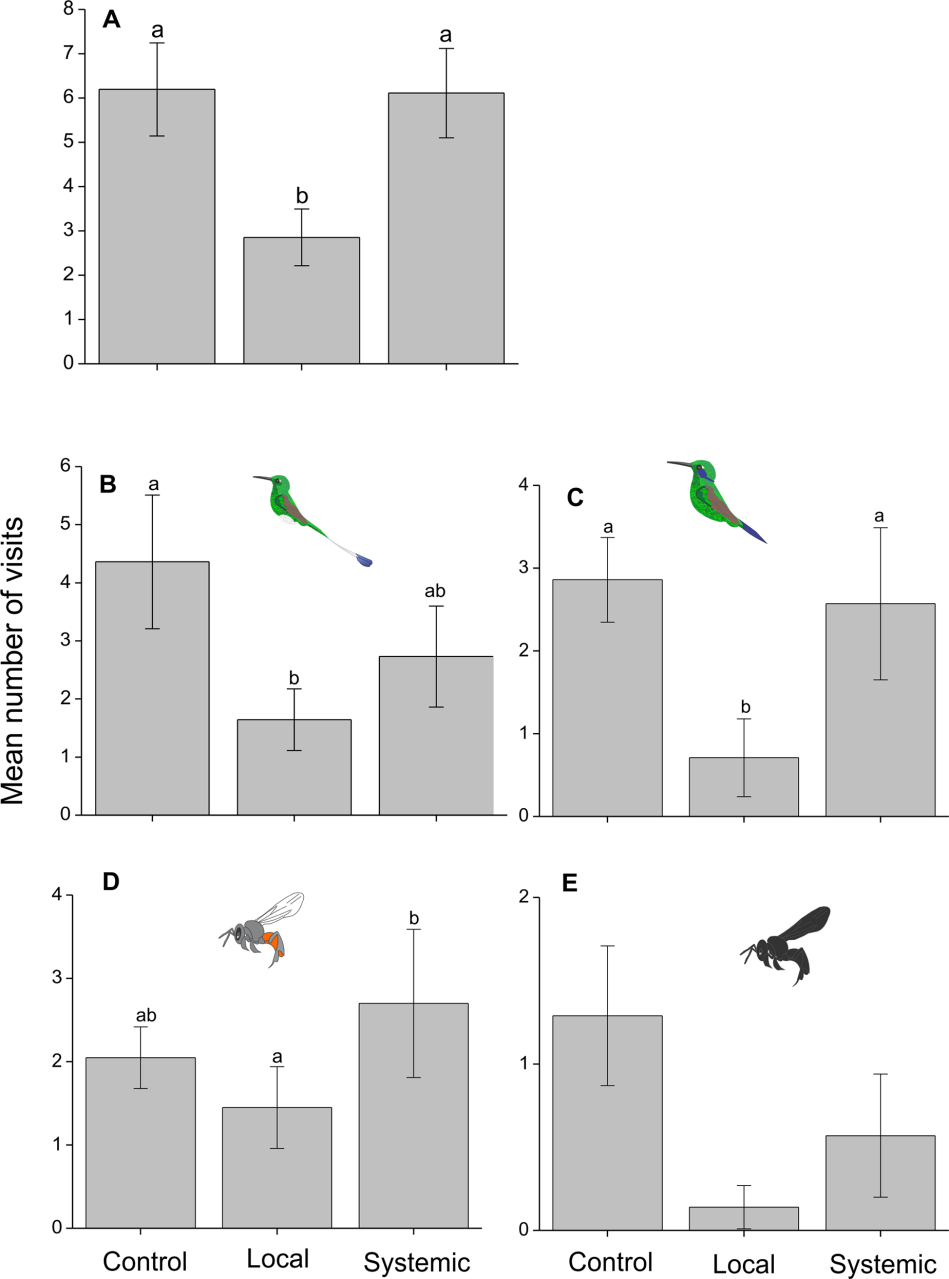
Mean number of visits of pollinators (+/-SE) to *Palicourea angustifolia* plants that have been locally or systemically exposed to herbivory by *Zeromastax selenesii* and non-exposed (control plants). **A.** All pollinator species combined **B.** *Ocreatus underwoodii*, **C.** *Colibri thalassinus*, **D.** *Trigona fulviventris* and **E.** *Paratrigona* sp. Treatments with a common letter are not significantly different (Tukey test, P > 0.05).

The average number of visits to flowers from all visitors combined was strongly affected by treatment, with the highest number of visits on control inflorescences, a roughly similar number on systemically-induced inflorescences, and 54% fewer visits on locally-induced inflorescences (Table 2, Fig 3A). The number of visits was not affected by the number of open flowers on the branch or the interaction between treatment and the number of open flowers (Table 2).

**Table 2:** Output of the generalized linear mixed models analyzing the effects of foliar herbivory treatment (local induction, systemic induction and control), number of open flowers and their interaction on the total number of visits to flowers, the total number of visitors, and the visits of individual species.

Response variable	Explanatory variables	χ^2^	df	p
Total number of visits				
	Treatment	44.27	2	<0.001
	Number of flowers	1.86	1	0.17
	Treatment x Number of flowers	2.4	2	0.3
Total number of visitors				
	Treatment	13.77	2	0.001
	Number of flowers	0.78	1	0.38
	Treatment x Number of flowers	0.16	2	0.92
Visits by *Ocreatus underwoodii*				
	Treatment	12.87	2	0.002
	Number of flowers	0.01	1	0.91
Visits by *Colibri coruscans*				
	Treatment	15.31	2	<0.001
	Number of flowers	5.29	1	0.002
Visits by *Trigona fulviventris*				
	Treatment	8.73	2	0.013
	Number of flowers	1.39	1	0.24
Visits by *Paratrigona* sp				
	Treatment	6.44	2	0.024
	Number of flowers	0.06	1	0.81

**Fig 4:**
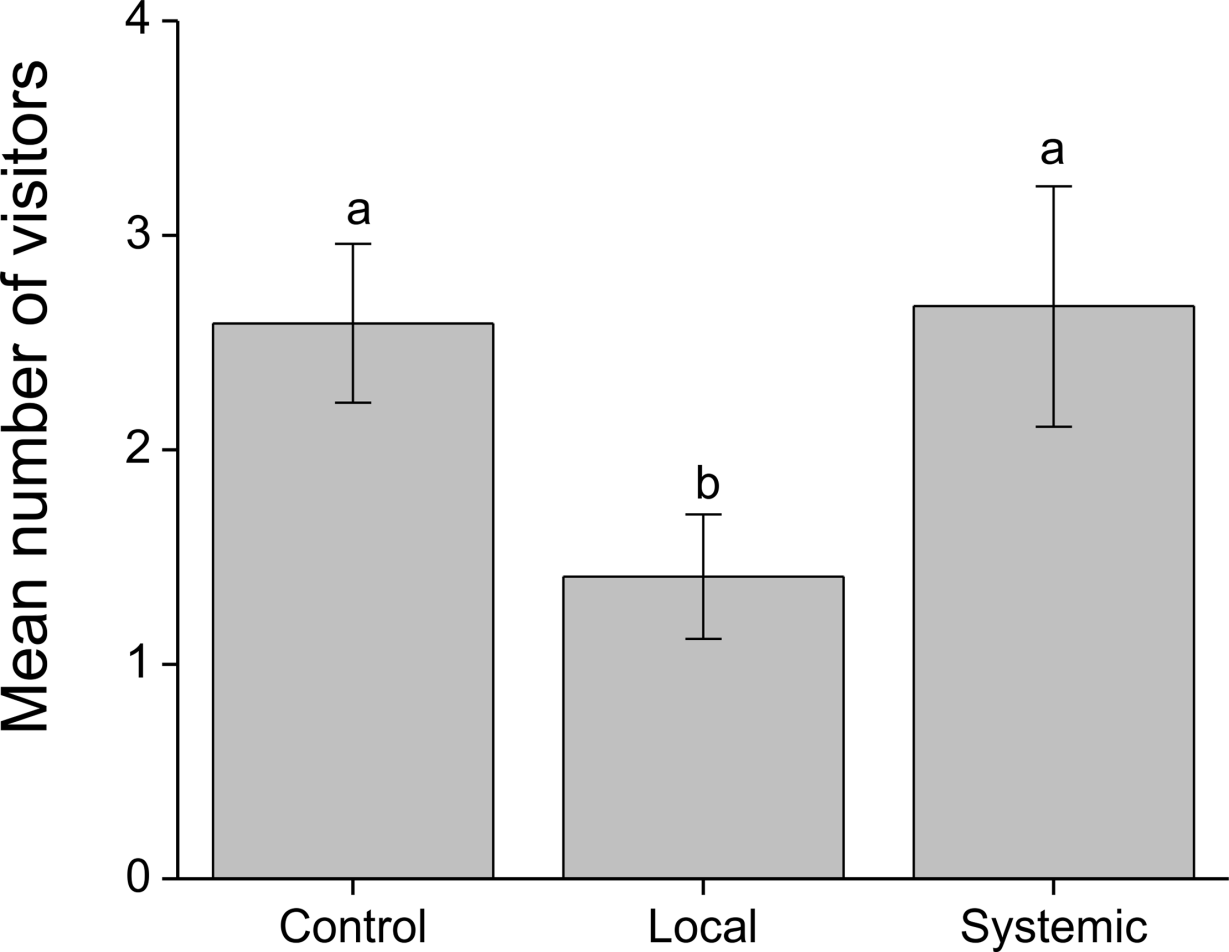
Mean number of visitors (+/-SE) to *Palicourea angustifolia* plants that have been locally or systemically exposed to herbivory by *Zeromastax selenesii* and non-exposed (control plants). Data are summed across all pollinator species. Treatments with a common letter are not significantly different (Tukey test, P > 0.05).

The average number of individual visitors to flowers was also strongly affected by treatment, with the highest number on control inflorescences, a similar number on systemically-induced inflorescences, and 46% fewer visitors on locally-induced inflorescences (Table 2, Fig 4). The number of visitors was not affected by the number of open flowers on the branch or the interaction between treatment and number of flowers (Table 2).

The most frequent floral visitors of *P*. *angustifolia* were hummingbirds (six species) and bees (five species, dominated by *T*. *fulviventris*), with some additional visits from one unidentified wasp species and one butterfly species (S1 Table). A Chi-square test for independence showed that the effect of treatment on the number of visits was dependent on the pollinator identity (Χ^2^ = 61.75, df = 360 24, P < 0.001) and thus further analyses were conducted separately for individual species. For *O*. *underwoodii* hummingbirds, there was a reduced number of visits on locally-induced branches relative to controls (Table 2, Fig 3B) and no effect of the number of flowers. For *C*. *coruscans* hummingbirds, there was a reduced number of visits on locally-induced branches relative to systemically-induced or controls (Table 2, Fig 3C) and a significant positive effect of the number of open flowers (Table 2). For *T*. *fulviventris* bees, there was a reduced number of visits on locally-induced branches relative to systemically-induced (Table 2, Fig 3D) and no effect of the number of flowers (Table 2). For *Paratrigona sp*. bees, there was an overall effect of treatment on the number of visits (Table 2, Fig 3E), but *post-hoc* tests showed only a marginal decrease in visitation to locally-induced branches relative to controls (*P* = 0.088). There was no effect of the number of flowers (Table 2).

### Effects of foliar herbivory on floral morphology and nectar rewards

**Fig 5:**
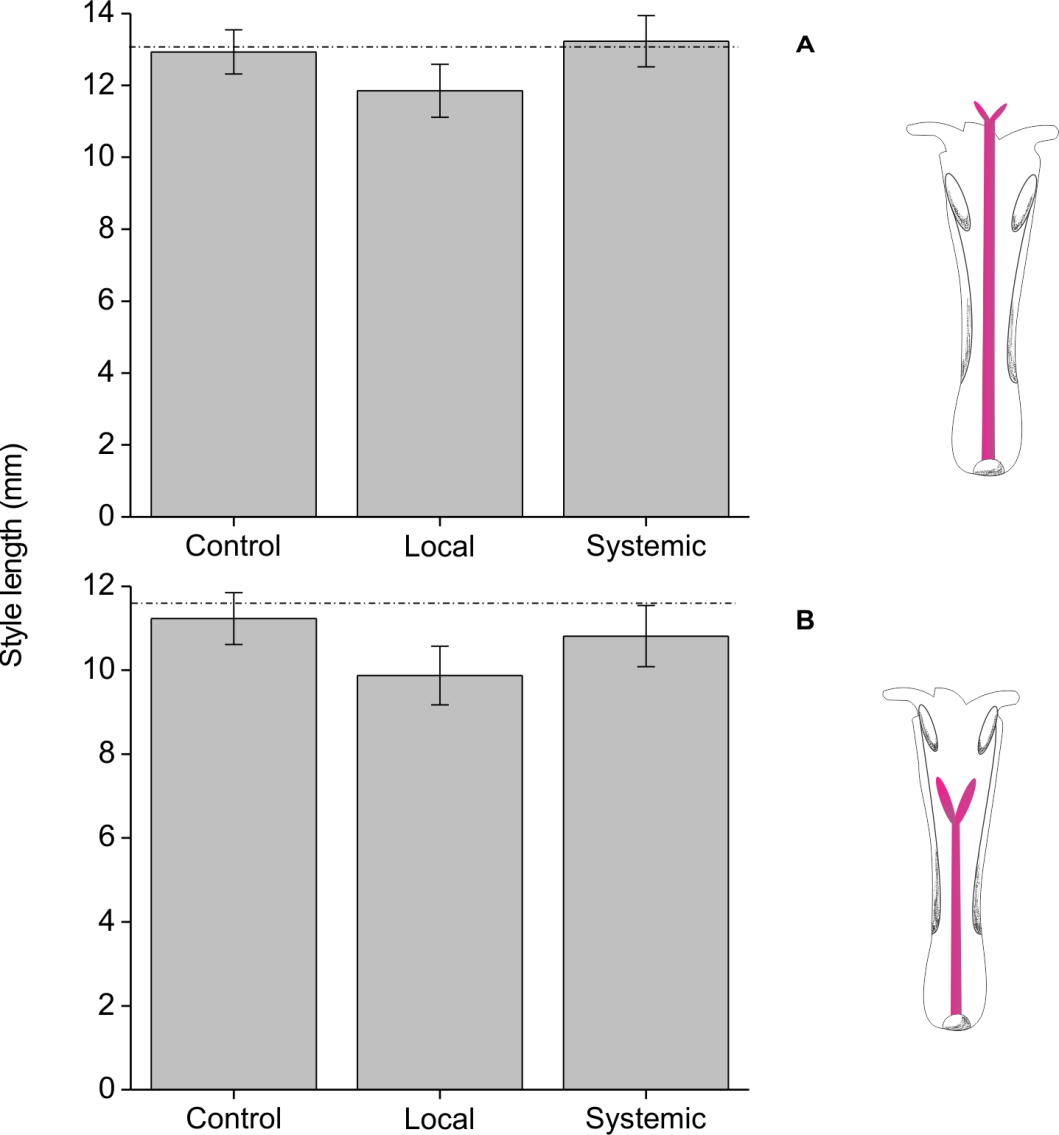
Length style (+/-SE) of *Palicourea angustifolia* plants that have been locally or systemically exposed to herbivory by *Zeromastax selenesii* and non-exposed (control plants). Local induction via foliar herbivory reduced style length in both pin (A) and thrum (B) flowers. Statistical analyses were conducted on the combined dataset for both floral morphs (see text) and post-hoc pairwise comparisons of style lengths among treatments showed that styles from locally-induced flowers were significantly shorter than those from systemically-induced flowers (P = 0.007) or control flowers (P = 0.004). Mean style lengths for each morph are shown separately here for illustrative purposes to allow comparison to the mean anther length (dotted horizontal lines) in control flowers of the opposite morph.

The only floral morphological trait affected by herbivory treatment was style length (Table 3, Fig 5). Styles were about 10% shorter on locally-induced branches than on systemically-induced branches or controls (Table 3; Fig 5). There were also differences between floral morphs (styles are longer on pin morphs), but no interaction between floral morph and treatment (Table 3). Corolla length and anther length were not affected by herbivory or by the interaction between herbivory and morph but were different between morphs (Table 3).

**Table 3:** Results from linear mixed effects models showing the effect of foliar herbivory treatment (local induction, systemic induction and control), and morph (pin or thrum) on floral morphology and nectar rewards of *Palicourea angustifolia*.

	Treatment x Morph			Treatment			Morph		
	*Χ*^*2*^	*df*	*P*	*Χ*^*2*^	*df*	*P*	*Χ*^*2*^	*df*	*P*
Corolla Length	0.66	2	0.72	1.17	2	0.56	6.20	1	**0.01**
Anther Length	0.61	2	0.74	5.00	2	0.08	6.20	1	**0.01**
Style Length	0.95	2	0.62	12.12	2	**0.002**	5.40	1	**0.02**
Nectar Volume	1.72	2	0.42	10.40	2	**0.006**	5.08	1	**0.02**
Nectar Concentration	3.70	2	0.16	7.53	2	**0.02**	0.16	1	0.69

**Fig 6:**
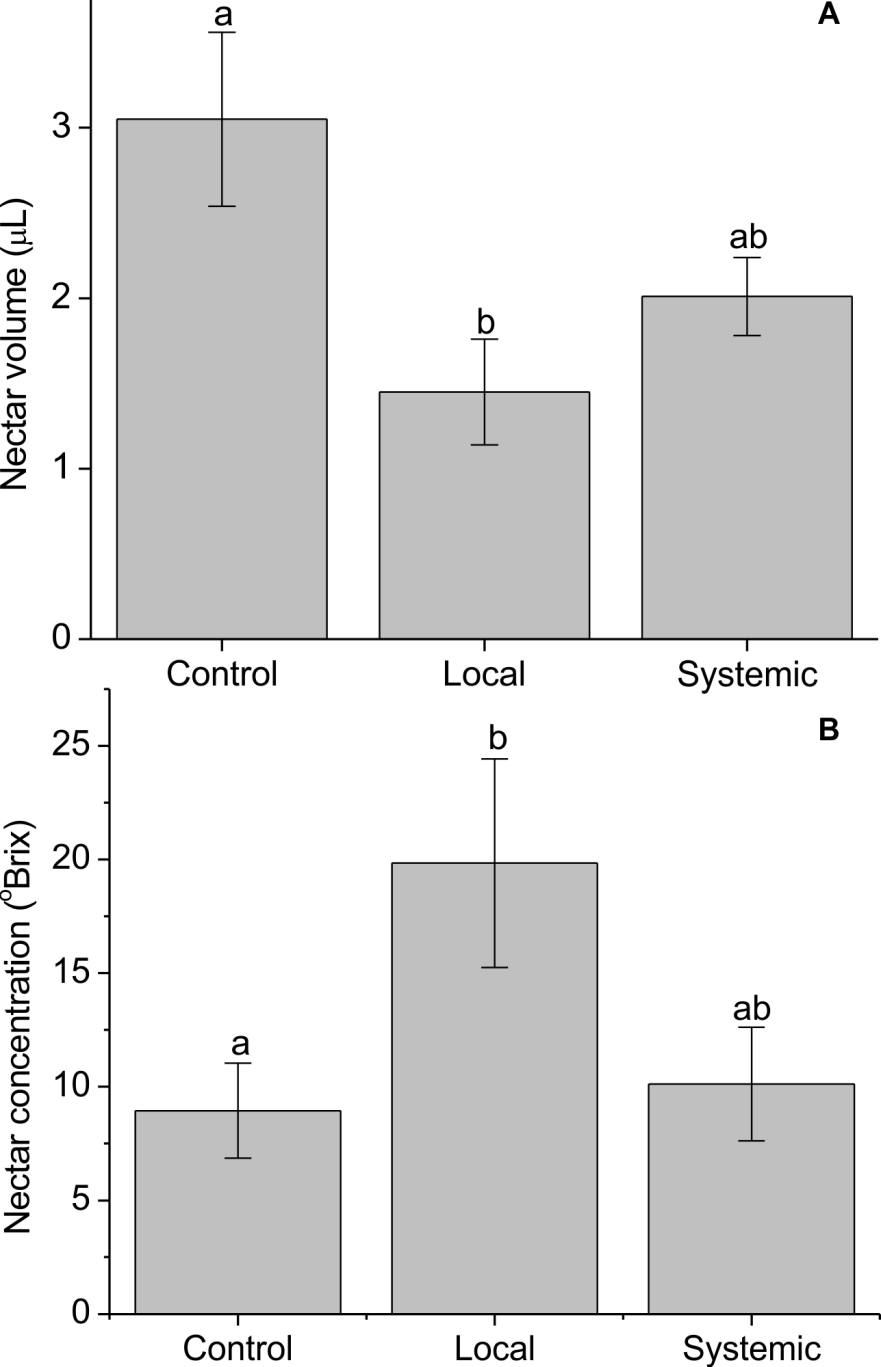
Nectar volume (A) and nectar concentration (B) (+/- SE) of *Palicourea angustifolia* plants that have been locally or systemically exposed to herbivory by *Zeromastax selenesii* and non-exposed (control plants). Treatments with a common letter are not significantly different (Tukey test, P > 0.05).

Nectar volume was negatively affected by herbivory treatment, with locally-induced branches producing the lowest average volumes of nectar (1.45 μl per flower), systemically-induced branches producing intermediate volumes (2.01 μl per flower) and control branches producing the highest volumes (3.05 μl per flower) (Table 3; Fig 6A). Nectar concentration was also affected, but in the opposite direction, with higher average nectar concentrations on locally-induced branches (19.84 ^o^Bx) compared to systemically-induced (10.12 ^o^Bx) or controls (8.95 ^o^Bx) (Table 3, Fig 6B).

### Effects of foliar herbivory on volatile emissions of flowers and leaves

**Fig 7:**
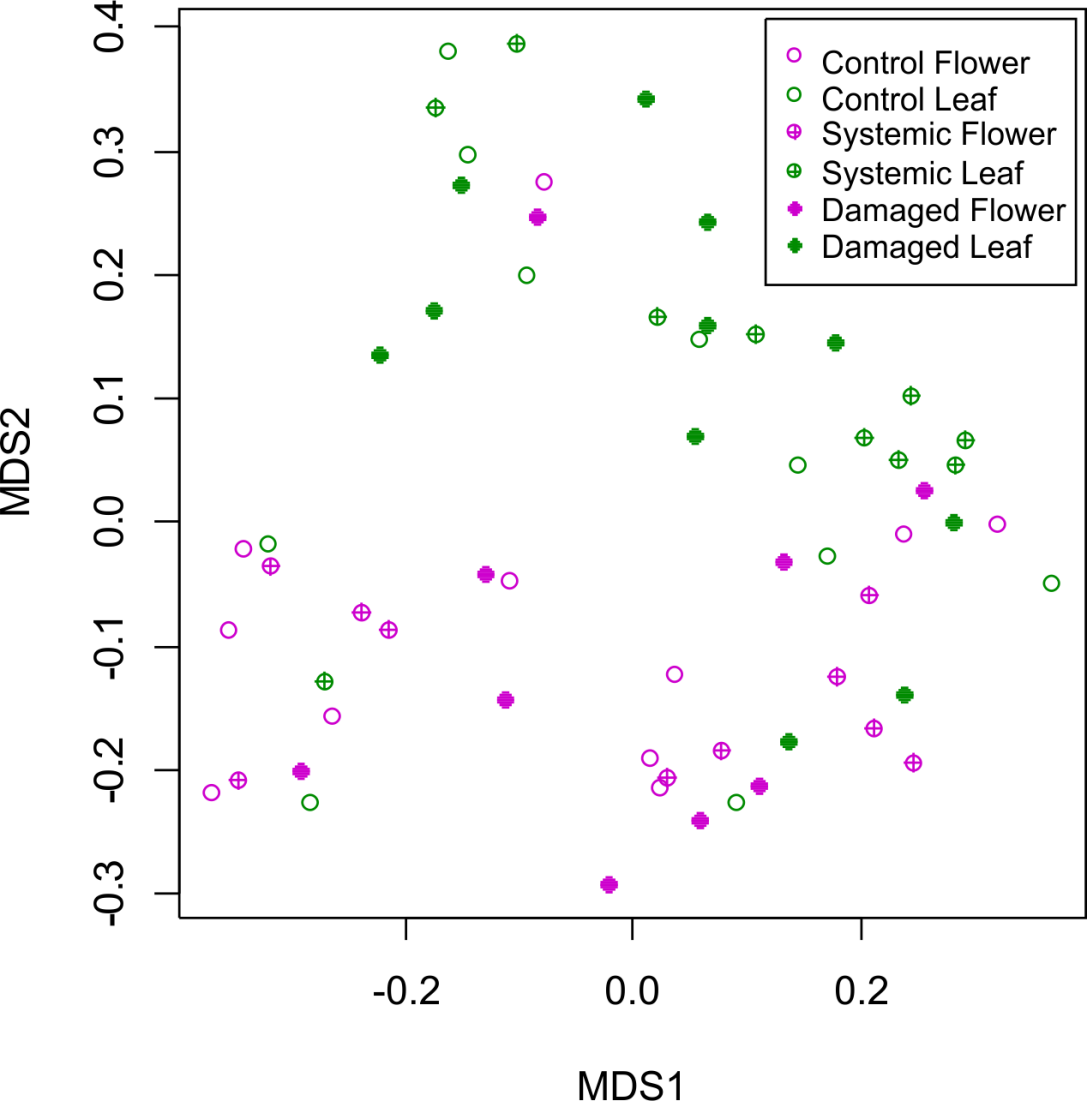
Multidimensional scaling plot (MDS) based on a random forest analysis showing similarity of *Palicourea angustifolia* volatile profiles among plant parts (leaves and flowers) and among foliar herbivory treatments (local induction, systemic induction and control).

Overall volatile emission profiles were clearly different between leaves and flowers, but there were no clear effects of foliar herbivory treatment (based on visual inspection of the random forest MDS plot; Fig 7). The RF classification model comparing leaves and flowers had a bootstrap estimate of prediction error of 0.23 and revealed five major compounds that were important in distinguishing the two groups. A MANOVA comparing concentrations of these compounds in leaves and flowers showed overall differences between the two organs (S2 Table).

The volatile blends from branches receiving different foliar herbivory treatments were not distinguishable based on the RF classification models. For the floral volatiles, the bootstrap estimate of prediction error was 0.73 (worse than a predicted random error rate of 0.66), and a MANOVA showed no differences among treatments for the three compounds ranked as most important by the RF (*F*_8,50_ = 1.34, *P* = 0.25). Similarly, for the leaf volatiles, the bootstrap estimate of prediction error was 0.68. However, a MANOVA comparing among treatments for the four compounds ranked as most important revealed significant overall differences (*F*_8,52_ = 2.50, *P* = 0.02), and follow-up ANOVAs revealed one unidentified compound that was marginally higher in control leaves compared to locally-induced leaves (ANOVA: *F*_2,28_ = 2.94, *P* = 0.07; post-hoc Tukey contrast between locally-induced and control: *P* = 0.06).

## Discussion

Plant responses induced by herbivore damage are a defense mechanism that can protect plants against further damage [39]. However, induced responses have also been shown to be ecologically costly in that they can reduce visits by pollinators and cause a reduction in seed set [7,8]. Here we contribute to this body of work by showing that herbivore-induced responses in a distylous tropical shrub can reduce seed set (Fig 2) and affect hummingbird as well as insect visitation to flowers (Figs 3 and 4), particularly those flowers that are directly adjacent to damaged leaves. We examined multiple mechanisms through which herbivore damage may be influencing the preferences and efficiency of pollinators and found that induced responses in plants can lead to several changes in floral rewards and morphology. We detected a localized decrease in nectar volume and increase in nectar concentration following damage (Fig 6), which may have contributed to the observed reduction in pollinator preference that we found in at least two hummingbirdsd and two bee species. We also detected a localized decrease in style length in both pin and thrum floral morphs (Fig 5), which may decrease pollination efficiency in a distylous plant where cross-pollination between morphs depends on precise placement of pollen on pollinator body surfaces and a morphological match between the styles and the anthers of the opposite morphs [28].

Our experiments on pollination mechanisms showed that *P*. *angustifolia* is an obligate outcrosser and requires animal pollination for reproduction (Table 1). Distylous plants often exhibit complete heteromorphic incompatibility, where only legitimate visits between different morphs result in pollination and fruit set [40–42]. In our system there is still partial compatibility among flowers from different individuals of the same morph, as has been reported for other species of *Palicourea* [43]. However, pollination was approximately four-fold more effective when pollen was moved between different morphs (Table 1). This reciprocal cross-pollination may be achieved primarily by hummingbirds in *Palicourea*, since pollen can be deposited precisely along hummingbird bills as they probe flowers for nectar and efficiently transferred to the stigma of the opposite morph [43]. Hummingbirds also visit numerous flowers in each foraging bout [43], whereas the main bee species we observed, *T*. *fulviventris*, rarely moves among individual plants and can sometimes act as a pollen and nectar thief rather than a legitimate pollinator [43,44]. Our data also suggest that fruit set in *P*. *angustifolia* is pollen limited; open-pollinated control plants in our study produced an average fruit set of only 45% (Fig 3), compared to an average fruit set of 88% across all intermorph crosses in our hand-pollinated plants (Table 1). Together, these results suggest that any reduction in pollinator visitation, particularly from hummingbirds, that occurs because of induced responses to herbivory is likely to have an important impact on the reproductive success of *P*. *angustifolia*.

Our results clearly showed that branches subjected to leaf herbivory produced a proportionally lower number of fruits relative to undamaged controls (Fig 2). The effects on locally-induced inflorescences were the strongest (a 68% reduction relative to controls), but there were also strong effects on systemically-induced inflorescences (a 29% reduction relative to controls). These results are in agreement with past work showing a negative relationship between leaf herbivory and fruit set in other species of Rubiaceae [14,43]. The negative effects of herbivory on fruit set may be due to a combination of physiological costs, which arise due to a direct reduction in resources available for fruit production following herbivore damage, and ecological costs, which arise due to altered interactions with the plant’s environment [8]. Both types of costs are commonly reported, but are often interdependent and difficult to disentangle experimentally [8]. Our results suggest that physiological costs of herbivory, manifested as changes in floral morphology and nectar rewards, can lead to ecological costs in terms of reduced visitation or efficiency, magnifying any negative effects on plant fitness that may occur due to reduced resource allocation to fruit development.

Results from our floral visitation experiments (Figs 3 and 4) show that hummingbirds as well as insect floral visitors can identify and discriminate against inflorescences on previously damaged branches. Visitation was reduced most strongly in locally-induced branches (Figs 3 and 4), which indicates that at least some of the chemical or physical changes in floral traits following damage are fairly localized on the plant. All the pollinator species that we could examine statistically were negatively affected by the induction treatments; however, different pollinator species did vary in the strength of their responses and in the relative deterrence of locally and systemically-induced flowers (S1 Table, Fig 3). Furthermore, trends in the pollinator species that we did not analyze statistically due to very low visitation rates were quite variable; in some cases, (e.g. *Colibri thalassinus*) visitation was even higher on induced branches than on controls (S1 Table). This variation could be due in part to differences among pollinator species in the complex combinations of visual, olfactory, or gustatory cues that they use to discriminate among flowers [45].

Changes in nectar volume and concentration were most likely to explain the observed changes in pollinator preference. Nectar volume has been shown to be a particularly important trait for determining hummingbird feeding preferences [46], and the pattern of nectar volumes among treatments (Fig 4) does concur with the pattern of hummingbird preferences (Fig 3). However, hummingbirds also prefer higher nectar concentrations within the range of concentrations typical in nectars [47], and it is unclear in our study why nectar concentration increased following herbivory and how it may have influenced hummingbird preferences. Herbivory has been shown to cause localized water stress [48,49], and one hypothesis is that herbivory reduced the quantity of water available for nectar production, reducing nectar volume and increasing its concentration as we observed in our study. There are also several other traits that may have influenced hummingbird and insect preferences that were not quantified in this study. To our knowledge, *P*. *angustifolia* has not previously been investigated phytochemically, but related species contain a diversity of potentially toxic secondary metabolites, including alkaloids and iridoid glycosides [50], and these classes of compounds can occur in nectar or pollen and may be induced in response to damage [1,51]. Further research, including chemical analyses and behavioral experiments with key pollinator species, would be necessary to fully understand how the complex changes in floral traits following damage may influence pollinator preference.

Another important mechanism through which herbivory may influence pollination success is by altering floral morphology in a way that affects the efficiency of pollen transfer. Our results showed that herbivory can cause a reduction in style length (Fig 5), similar to results from previous work [21]. This effect has previously been interpreted as a means to decrease anther-stigma separation and allow more self-pollination in herbivore-damaged plants that are less likely to be visited by pollinators [21]. However, the adaptive function of this proposed mechanism depends on flowers that are homomorphic and self-compatible. The consequences of reduced style length in an obligate outcrossing distylous plant such as *P*. *angustifolia* are quite different. Shorter styles would result in reduced anther-stigma distance in pin morphs and increased anther-stigma distance in thrum morphs, but this change would have limited effects on fertilization and reproductive output in a self-incompatible plant. More importantly, a change in style length would lead to a mis-match in lengths between styles and the anthers of the opposite morphs (Fig 5), thereby breaking down the reciprocal pollen transfer mechanism that is thought to be the primary evolutionary advantage of distyly [28]. Thus, in the case of *P*. *angustifolia* and other distylous plants, any reduction in style length very likely represents an ecological cost of defense.

Although previous reports have shown significant differences in the volatile profiles of flowers and leaves before and after herbivory [4,22,23], we did not detect such differences in the volatile profiles of leaves or flowers of *P*. *angustifolia*. However, we did clearly detect different compounds in the leaves and flowers of *P*. *angustifolia*, refuting the misconception that flowers of ornithophilous plants produce no or very low quantities of volatiles that are not detectable by GCMS [52]. There are two ways in which we could interpret our results. One is that the reduction in flower visitation is not dictated by a change in the volatile profile of the plant after herbivory. The other explanation is that the volatiles that mediate the interaction between *Palicourea* and its pollinators are present in minor quantities that we were not able to detect with our methodology.

Overall, this study showed that foliar herbivory can alter interactions with hummingbird and insect pollinators and lead to ecological costs of defense in a tropical distylous plant. Changes in hummingbird and insect visitation following herbivory were likely mediated by a combination of changes in floral traits, including changes in flower morphology and changes in nectar volume and nectar concentration. These results emphasize the potential for complex interactions between plant antagonists and mutualists and the importance of studying these interactions simultaneously to gain a complete picture of the costs and benefits of plant responses to herbivory.

## Supporting information

S1 File Differences in flower morphology and nectar production between thrum (T) and pin (P) flowers in *Palicourea angustifolia*. (DOCX)

S1 Table Summary of pollinator visitation to inflorescences on different branch treatments. Number of replicate plant pairs (of N = 26 total) in which a visitor was observed on any of the treatments. Statistical analyses were conducted only for taxa that were observed in five or more replicates.
(DOCX)

S2 Table Volatile compounds in *Palicourea angustifolia* with differences in concentration in flowers and leaves. (DOCX)
