# Supplementary material for: Leaf herbivory imposes fitness costs mediated by hummingbird and insect pollinators
Source: PLoS One. 2017 Dec 6;12(12):e0188408. doi: 10.1371/journal.pone.0188408 (PMC5718403; doi:10.1371/journal.pone.0188408)
Supplement: S1 File — (DOCX) [file pone.0188408.s001.docx]

**S1 File**

Details on floral morphology and nectar reward of *Palicuoure angustifolia*

**Methods**

Preliminary observations of our study population showed that some plants have a large style and short stamens (pin) while others have a short style and large stamens (thrum), suggesting that *P. angustifolia* presented distyly, a phenomenon common in this genus. We were interested in confirming the presence of distyly in our study population and in determining if distyly would affect the nectar reward for floral visitors. To confirm that there is a morph variation we performed a morphometric study with 30 plants, 15 pin plants and 15 thrum plants. On each of those plants we measured 1) style length (from the base of the corolla to tip of the stigma), 2) stamen length (from the base of the corolla to the tip of the anthers), 3) anther length, and 4) distance between tip of the stigma and tip of the anthers. Additionally, we evaluated the nectar production in both pin and thrum flowers. In order to take nectar measurement we bagged one inflorescence per plant a day before taking the measurements. Nectar was taken with 2µL capillary tubes (Drummond Scientific Company, Broomall, PA) and its volume estimated by measuring the length of the nectar column in the capillary tube and multiplying it by the diameter. The sugar content in the nectar was measured using a handheld refractometer (Reichert Digital Brix/RI-Chek Refractometer). Given the limited nectar volume to take a measurement we diluted the sample in 0.1 mL of distilled water. To analyze if there were differences in the floral measurements like anther length, style length and distance between stigma and anther, nectar concentration and volume between pin and thrum plants we performed student`s t-tests. To estimate the ratio between the two morphs in the population we walked a 500m transect along a trail and quantified the number of plants of each morph.

## Results

Our results show that *P. angustifolia* clearly presents distyly. The abundance of both morphs was relatively equal. Out of the 136 plants surveyed 72 (52.9%) plants were thrum and 64 (47.1%) plants were pin. Corollas and stamens in thrum flowers were longer (t=2.39, p=0.021 and t=3.13, p=0.003, S1 Fig. A and B, respectively), while style length and anther-stigma distance were shorter (t=10.67, p<0.001, and t=7.28, p<0.001, S1 Fig C and D. respectively). Anther length was the same in both morphs (t=0.21, p=0.83) and we did not find differences in both nectar volume and concentration between thrum and pin flowers (t= -1.58, p=0.12 and t= 0.09, p=0.92, respectively).

B

A

Stamen length (mm)

Corolla length (mm)

C

D

T P

T P

Anther-Stigma distance (mm)

Style length (mm)

**S1 figure.** Differences in flower morphology between thrum (T) and pin (P) flowers in *P. angustifolia* (mean±SE). Represented of corolla length (A), Stamen length (B), style length (C) and anther-stigma distance (D).
