# Supplementary material for: Leaf herbivory imposes fitness costs mediated by hummingbird and insect pollinators
Source: PLoS One. 2017 Dec 6;12(12):e0188408. doi: 10.1371/journal.pone.0188408 (PMC5718403; doi:10.1371/journal.pone.0188408)
Supplement: S1 Table — Number of replicate plant pairs (of N = 26 total) in which a visitor was observed on any of the treatments. Statistical analyses were conducted only for taxa that were observed in five or more replicates. (DOCX) [file pone.0188408.s002.docx]

| Pollinator | Control | Local Induction | Systemic Induction | Number of Reps.^a^ |
| --- | --- | --- | --- | --- |
| **Hummingbirds:** |  |  |  |  |
| *Ocreatus underwoodii* | 48 | 18 | 28 | 11 |
| *Colibri coruscans* | 17 | 5 | 18 | 6 |
| *Colibri thalassinus* | 4 | 10 | 13 | 4 |
| *Clorostilbon portmanni* | 2 | 4 | 12 | 4 |
| *Heliodoxa rubinoides* | 9 | 4 | 6 | 2 |
| *Boissonneaua flavescens* | 5 | 0 | *4* | *1* |
| **Total Hummingbirds** | 85 | 41 | *81* | *19* |
| **Bees:** |  |  |  |  |
| *Trigona fulviventris* | 41 | 29 | 54 | 20 |
| *Paratrigona* | 9 | 1 | 4 | 7 |
| *Apis mellifera* | 7 | 1 | 7 | 3 |
| *Bombus* | 0 | 5 | 8 | 2 |
| *Thygater* | 3 | 0 | 3 | 1 |
| **Total Bees** | 60 | 36 | 76 | 12 |
| **others:** |  |  |  |  |
|  | 19 | 0 | 4 | 11 |
|  |  |  |  |  |
| **Total Visits** | 164 | 77 | 161 | 27 |
|  | | | | |

**S1 Table. Summary of pollinator visitation to inflorescences on different branch treatments.** ^a^ Number of replicate plant pairs (of N = 26 total) in which a visitor was observed on any of the treatments. Statistical analyses were conducted only for taxa that were observed in five or more replicates.
