# Supplementary material for: Leaf herbivory imposes fitness costs mediated by hummingbird and insect pollinators
Source: PLoS One. 2017 Dec 6;12(12):e0188408. doi: 10.1371/journal.pone.0188408 (PMC5718403; doi:10.1371/journal.pone.0188408)
Supplement: S2 Table — (DOCX) [file pone.0188408.s003.docx]

| **S2 Table.** **Volatile compounds in *Palicourea angustifolia* with differences in concentration in flowers and leaves**  Volatiles collected from flowers and leaves of *P. angustifolia* were collected using dynamic head space method. The samples were analyzed by GC-MS. To examine the differences in volatiles production between flowers and leaves we used a random forest analysis with 200 bootstrap iterations of each analysis to select compounds that best distinguished among groups. Compounds that were selected in greater than 20% of bootstrapped models were retained for use in MANOVAs comparing the concentrations of the selected compounds among groups. Where significant overall differences among groups were detected, we followed the MANOVAs with ANOVAs comparing the concentrations of individual compounds among groups (F and p values presented just for these compounds). Tentative identification was carried using the NIST mass spectra data bases. | | | | | | |
| --- | --- | --- | --- | --- | --- | --- |
| Retention time | | Tentative ID | Kovats index | F 4.65 | df 6,54 | p <0.001 |
| 15.01 | | Decyl ether | 1214.76 |  |  |  |
| 8.89 | | O-xylene | 854.07 |  |  |  |
| 26.06 | | Furaltadone | 1893.95 |  |  |  |
| 26.09 | | Unknown A | 1895.82 |  |  |  |
| 12.23 | | Unknown B | 1026.43 | 16 | 1,59 | <0.001^a^ |
| 23.46 | | 1-Pentatriacontanol | 1731.65 |  |  |  |
| 13.53 | | Unknown C | 1110.08 |  |  |  |
| 17.37 | | 3-p-Menthol | 1372.25 |  |  |  |
| 26.24 | | N-Dimethylaminomethyl-tert.-butyl-isopropylphosphine | 1909.63 |  |  |  |
| 8.73 | | P-xylene | 847.18 |  |  |  |
| 8.55 | | Unknown D | 839.43 |  |  |  |
| 25.26 | | Unknown E | 1844.01 |  |  |  |
| 25.24 | | Unknown F | 1842.76 |  |  |  |
| 23.34 | | N,N-Dimethyl-1-undecanamine | 1724.16 |  |  |  |
| 24.16 | | Isopropyl myristate | 1775.34 |  |  |  |
| 16.5 | | Unknown G | 1316.62 |  |  |  |
| 21.03 | | Nerylacetone | 1590.85 |  |  |  |
| 12.78 | | Unknown H | 1061.37 |  |  |  |
| 11.6 | | Beta cymene | 988.48 |  |  |  |
| 18.14 | | Unknown I | 1420.28 |  |  |  |
| 13.78 | | Unknown J | 1127.82 |  |  |  |
| 20.14 | | Unknown K | 1539.32 |  |  |  |
| 18.1 | | 3,5,24-Trimethyltetracontane | 1417.86 |  |  |  |
| 18.33 | | Alpha terpineol | 1431.74 |  |  |  |
| 8.32 | | Beta phellandrene | 829.53 |  |  |  |
| 10.29 | | Cineole | 917.93 |  |  |  |
| 23.99 | | Diphenyl ether | 1764.73 |  |  |  |
| 14.72 | | P-menthonef | 1194.54 |  |  |  |
| 16.81 | | Nerol | 1336.45 | 5.7 | 1,59 | 0.02^a^ |
| 27.22 | | 1-chlorooctadecane | 2023.32 |  |  |  |
| 7.93 | | Alpha pinenef | 812.74 |  |  |  |
| 20.65 | | P-allylanisole | 1568.85 |  |  |  |
| 18.45 | | 2-Methylenecholestan-3-ol | 1438.99 |  |  |  |
| 14.43 | | 3-octenol | 1173.95 | 3.5 | 1,59 | 0.07^a^ |
| 21.59 | | Unknown M | 1622.78 |  |  |  |
| 13.5 | | 3-(3-methylbutyl) cyclopentene | 1107.95 | 4.5 | 1,59 | 0.04^a^ |
| 9.38 | | Alpha phellandrene | 875.16 |  |  |  |
| 20.41 | | 2-Methylenecholestan-3-ol | 1554.95 |  |  |  |
| 18.96 | | Benzyl acetate | 1469.76 |  |  |  |
| 17.29 | | 1-Heneicosyl formate | 1367.14 |  |  |  |
| 19.82 | | Methyl salicylatef | 1520.79 |  |  |  |
| 25.07 | | 1,16-Dichlorohexadecane | 1832.15 |  |  |  |
| 22.66 | | Unknown N | 1683.40 | 3.8 | 1,59 | 0.06^b^ |
| 20.71 | | (Z)-9-Tricosene | 1572.32 |  |  |  |
| 17.96 | | Unknown O | 1409.41 |  |  |  |
| 21.39 | | Unknown P | 1611.44 |  |  |  |
| ^a^ found in higher concentration in flowers than leaves  ^b^ found in higher concentration in leaves than flowers | | | | | | |
|  | | | | | | |

The kovats index was calculated by comparing retention times with an alkane standard solution (C_8_-C_20,_ fluka 04070 Sigma-Aldrich Co), using the formula:

$$I=100\left[ n+\left( N-n \right)\frac{T_{ra}-T_{rn}}{T_{rN}-T_{rn}} \right]$$

- *I*: Kováts index
- *T_rn_* is the retention time of the alkane before
- *T_rN_* is the retention time of the alkane after
- *T_ra_* is the retention time of the compound that we want to calculate the Kovats Index
- *n* number of carbons of the alkane after
- *N* number of carbons of the alkane before
